# DEEPsc: A Deep Learning-Based Map Connecting Single-Cell Transcriptomics and Spatial Imaging Data

**DOI:** 10.3389/fgene.2021.636743

**Published:** 2021-03-23

**Authors:** Floyd Maseda, Zixuan Cang, Qing Nie

**Affiliations:** ^1^Department of Mathematics, University of California, Irvine, Irvine, CA, United States; ^2^The NSF-Simons Center for Multiscale Cell Fate Research, University of California, Irvine, Irvine, CA, United States; ^3^Department of Developmental and Cell Biology, University of California, Irvine, Irvine, CA, United States

**Keywords:** spatial gene expression atlas, scRNA-seq data, spatial information imputation, deep learning, metric learning, comprehensive evaluation metric

## Abstract

Single-cell RNA sequencing (scRNA-seq) data provides unprecedented information on cell fate decisions; however, the spatial arrangement of cells is often lost. Several recent computational methods have been developed to impute spatial information onto a scRNA-seq dataset through analyzing known spatial expression patterns of a small subset of genes known as a reference atlas. However, there is a lack of comprehensive analysis of the accuracy, precision, and robustness of the mappings, along with the generalizability of these methods, which are often designed for specific systems. We present a system-adaptive deep learning-based method (DEEPsc) to impute spatial information onto a scRNA-seq dataset from a given spatial reference atlas. By introducing a comprehensive set of metrics that evaluate the spatial mapping methods, we compare DEEPsc with four existing methods on four biological systems. We find that while DEEPsc has comparable accuracy to other methods, an improved balance between precision and robustness is achieved. DEEPsc provides a data-adaptive tool to connect scRNA-seq datasets and spatial imaging datasets to analyze cell fate decisions. Our implementation with a uniform API can serve as a portal with access to all the methods investigated in this work for spatial exploration of cell fate decisions in scRNA-seq data. All methods evaluated in this work are implemented as an open-source software with a uniform interface.

## Introduction

While cells of a biological system have access to the same genetic blueprint, they navigate through different developmental paths toward various cell fates. These diverse fate programs of cells are controlled by their own states, interactions with spatially neighboring cells, and other environmental cues (Guo et al., 2010). To decipher the processes of cell fate acquisitions, observations of the transcriptomics with single-cell resolution in spatial context are desired. The advent of sophisticated single-cell RNA sequencing (scRNA-seq) techniques now allows investigation of the transcriptomic landscape of tens of thousands of genes across tissues at the resolution of individual cells (Rosenberg et al., 2018; Svensson et al., 2018). However, a drawback to scRNA-seq methods is the necessity of dissociating the sample in question, thereby destroying any spatial context which can be crucial to the understanding of cellular development and dynamics (Yuan et al., 2017). In current common workflows of scRNA-seq data analysis, unsupervised clustering of cells is carried out, followed by identifying marker genes associated with each cell cluster (Luecken and Theis, 2019). While the list of marker genes for each cell cluster can be screened for genes associated with known spatial regions to estimate the spatial origin of the cluster, the spatial arrangement of individual cells remains unclear (Kiselev et al., 2019; Luecken and Theis, 2019). Several existing methods attempt to impute a pseudospatial or pseudotemporal axis onto the data (Joost et al., 2016; Puram et al., 2017; Pandey et al., 2018; Wang et al., 2019); however, little related to physical space is immediately discernible from scRNA-seq data alone.

The loss of spatial information in scRNA-seq data can be partially mitigated by referring to spatial staining data (Sprague et al., 2006; Fowlkes et al., 2008). Another promising solution is the emerging spatial transcriptomics methods such as osmFISH (Codeluppi et al., 2018), MERFISH (Moffitt et al., 2018), seqFISH (Shah et al., 2016), seqFISH+ (Eng et al., 2019), STARmap (Wang et al., 2018), and Slide-seq (Rodriques et al., 2019) that obtain *in situ* spatial expression patterns. Compared to scRNA-seq, current spatial techniques often cover fewer cells or genes or with a suboptimal resolution and depth. It is therefore a trending theme to combine the strengths of both methods to achieve a high coverage and individual-cell resolution while retaining the spatial arrangement (Yuan et al., 2017; Kiselev et al., 2019). Due to these differences among the scRNA-seq and spatial techniques, and biological systems, it is challenging to derive a generally applicable computation method to integrate the two kinds of data.

Several recent computational methods have been developed to impute spatial data onto existing scRNA-seq datasets through analyzing known spatial expression patterns of a small subset of genes, termed a “spatial reference atlas.” Seminal methods were developed independently by Achim et al. (2015) and Satija et al. (2015) and were applied to the *Platynereis dumerilii* brain and zebrafish embryo, respectively, using binarized reference atlases derived from *in situ* hybridization (ISH) images. DistMap, another method that uses a binarized ISH-based reference atlas, was developed by Karaiskos et al. (2017) and applied to the *Drosophila* embryo. Achim et al. (2015) use an empirical correspondence score between each cell-location pair based on the specificity ratio of genes. Satija et al. (2015) (Seurat v1) fits a bimodal mixture model to the scRNA-seq data and then projects cells to their spatial origins using a probabilistic score. DistMap applies Matthew’s correlation coefficients to the binarized spatial imaging and scRNA-seq data to assign a cell-location score (Karaiskos et al., 2017). Several methods have also been developed which use spatial reference atlases directly measuring the RNA counts that are comparable to scRNA-seq data without binarization (Peng et al., 2016; Halpern et al., 2017). More recently, computational methods have been developed for imputing gene expression in spatial data (Lopez et al., 2019), transferring cell type label from scRNA-seq data to spatial data (Zhu et al., 2018; Dries et al., 2019; Andersson et al., 2020), *de novo* spatial placement of single cells (Nitzan et al., 2019), and inferring spatial distances between single cells (Cang and Nie, 2020).

In addition to the methods designed specifically for integrating spatial data and scRNA-seq data, other computational methods have been developed recently for general data integration. Such methods focus on the general task of integrating RNA sequencing datasets obtained from the same biological system through different technologies, *in situ* data being one possibility among many, into one large dataset offering a more complete description of the system under study. These methods include newer versions of Seurat (Butler et al., 2018; Stuart et al., 2019), LIGER (Welch et al., 2019), Harmony (Korsunsky et al., 2019), and Scanorama (Hie et al., 2019) which are mainly based on correlation analyses and matrix factorizations. Another more specific task is to transfer high-level information such as cell types between datasets. Many machine learning- and deep learning-based methods have been developed for this task by formulating a supervised learning problem with the high-level information being the target (Kiselev et al., 2018; Lieberman et al., 2018; Lopez et al., 2018; Wagner and Yanai, 2018; Tan and Cahan, 2019; Boufea et al., 2020; Hu et al., 2020; Ma and Pellegrini, 2020).

Since the spatial characteristics of different biological systems could be significantly different, we aim to develop a system-adaptive method specifically designed for imputing spatial information onto scRNA-seq data. To this end, unlike other spatial integration methods that use predefined algorithms for computing scores, we learn a specialized correspondence score between cells and locations for a given biological system. This can then be regarded as a general metric learning task (Kulis, 2013). In addition to linear methods that learn a pseudometric (Weinberger and Saul, 2009), there has been increasing interest in applying deep learning to metric learning (Kaya and Bilge, 2019; Chicco, 2020). These methods are mostly designed for cases where the pair of data points to be compared are in the same space. Though the common genes from the spatial data and scRNA-seq data are used here, directly treating them as in the same space may cause inaccuracy due to differences in the original datasets such as scaling and noise.

Here we develop a system-adaptive deep learning-based method (DEEPsc) for imputing spatial data onto scRNA-seq data. A DEEPsc network accepts a low-dimensional feature vector corresponding to a single position in the spatial reference atlas along with a corresponding feature vector of the gene expression of a single cell and returns a likelihood the input cell originated from the input position. The network is trained and validated using positions in the spatial reference atlas as simulated scRNA-seq data. The network is also validated through the task of predicting the scRNA-seq data from the spatial reference atlas or the other way around. In addition, we implemented several strong baseline methods using different norms and linear metric learning for benchmark comparison. We further develop a comprehensive measure, which was previously lacking, for evaluating how well a given method maps scRNA-seq data to known spatial origins, called a performance score. This score contains three components that measure the accuracy, precision, and robustness of a method, respectively. Using this score on four biological systems, we show that DEEPsc maintains a comparable accuracy to four existing methods while achieving a better balance between precision and robustness.

## Results

### A Deep-Leaning Based Method to Connect scRNA-seq Datasets and Spatial Imaging Data

Given any spatial reference atlas consisting of binary or continuous gene expression levels for a biological system on a set of locations with known spatial coordinates, and a scRNA-seq dataset consisting of binary or continuous gene expression levels for the same biological system, we introduce a **D**eep-learning based **E**nvironment for the **E**xtraction of **P**ositional information from **sc**RNA-seq data (DEEPsc) to impute the spatial information onto the scRNA-seq data.

**FIGURE 1:**
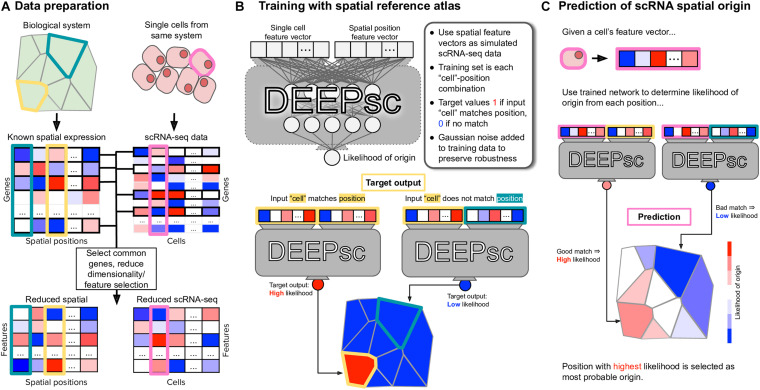
The general workflow of training and implementing DEEPsc. **(A)** Given a spatial reference atlas of gene expression levels for some biological system and a scRNA-seq dataset, genes common to both are selected, and dimensionality of the data is reduced (e.g., by PCA, UMAP). Each spatial position in the reference atlas and each cell in the scRNA-seq dataset is associated with a feature vector in the reduced space. **(B)** The DEEPsc architecture takes as input the feature vectors of one single cell and one spatial position, returning a likelihood between 0 (low likelihood) and 1 (high likelihood) that the cell originated from the spatial position. A DEEPsc network is trained using the spatial position feature vectors as simulated scRNA-seq data. The target output is a 1 (high likelihood of origin) if the simulated input cell matches the input position, and 0 (low likelihood of origin) if they do not match. **(C)** Once the DEEPsc network is sufficiently trained, a feature vector associated with a cell in the scRNA-seq dataset can be fed into the network with each spatial position individually. The resulting likelihoods are displayed as a heatmap depicting the likelihood of origin of the cell from each position. The position with the highest likelihood is chosen as the origin of the cell. This process is repeated for each cell in the scRNA-seq dataset.

In DEEPsc, we first select a common set of genes from the reference atlas and scRNA-seq data, then perform dimensionality reduction via principal component analysis (PCA) on the reduced reference atlas to shorten training time (Figure 1A). The scRNA-seq data is then projected into the same PCA space on which we learn a metric for comparison between cells and spatial positions. The DEEPsc network accepts a concatenated feature vector for a single cell and a single position and returns a likelihood the input cell originated from the input position. The network contains two fully connected hidden layers with *N* nodes each, where *N* is the number of principal components kept from PCA, and a single node in the output layer. Sigmoid activation functions are applied to each node, including the output node, so that the resulting output is in [0,1] and can be interpreted as a likelihood that the input cell originated from the input spatial position. To train the DEEPsc network, we use the spatial position feature vectors as simulated scRNA-seq data for comparison (Figure 1B). Each simulated cell is compared pairwise with every position in the spatial reference atlas; if the simulated cell is an exact match to a given position, the target output is 1 (a high likelihood of origin), and if the simulated cell and chosen position are not an exact match, the target output is 0 (a low likelihood of origin). Training is terminated when the error on a randomly chosen validation set is no longer improving.

After training the DEEPsc network, a feature vector associated with an actual cell from the scRNA-seq data is fed in as input and compared to each position in the reference atlas individually. We display the results as a heatmap on the schematic diagram of the biological system, choosing the spatial position with the largest likelihood of origin according to DEEPsc as the determined origin of the cell. This process is repeated for each cell in the scRNA-seq dataset to assign spatial origins of all cells (Figure 1C).

### Quantifying Spatial Mapping Performance

Each of the highlighted methods to impute spatial data onto scRNA-seq data, including DEEPsc, can be essentially boiled down to the following: For some tissue with a well-defined standard spatial structure, given known binary or continuous expression levels of *G* genes at each of *P* spatial locations (the reference atlas), calculate a correspondence score, *S*, of how similar each of *C* cells in an scRNA-seq dataset is to each of the *P* positions in the atlas. That is, define a function, *S*:[0,1]*^G^*×[0,1]*^G^*→[0,1], such that *S*(*c*_*i*_,*p*_*j*_);*i* = 1,2,…,*C*;*j* = 1,2,…,*P*; which describes the likelihood that cell *c*_*i*_ originated from position *p*_*j*_, based on the similarity of the expression vectors of the cell and position.

To quantify how well a given method performs for a given spatial reference atlas, we use the reference atlas itself as simulated single cell data; that is, we generate a simulated scRNA-seq dataset with *C=P* cells, each an exact copy of a reference atlas position. This allows us to treat the simulated scRNA-seq data as having a known spatial origin, against which we can compare the output of each method. We define a system-adaptive, comprehensive performance score, consisting of three penalty terms: accuracy, which determines whether or not the known spatial origin was given a high likelihood of origin; precision, which determines whether or not other locations were given low likelihoods of origin; and robustness, which determines how sensitive a mapping method is to random noise in the input data. Each penalty term is represented by a number in [0,1], with 0 being no penalty and 1 being a worst-case scenario. The performance score is defined as $E=\frac{1}{P}\,\sum_{i=1}E_{i}$, where

$$E_{i}=1-\frac{1}{3}\,\left(\underset{A\,c\,c\,u\,r\,a\,c\,y}{\underbrace{1-S_{i.i}}}+\left|\underset{P\,r\,e\,c\,i\,s\,i\,o\,n}{\underbrace{\frac{1-\sum_{j=1}^{P}S_{i,j}}{P-1}}}\right|+\underset{R\,o\,b\,u\,s\,t\,n\,e\,s\,s}{\underbrace{{\left(1-\sigma^{*}\right)}^{4}}}\right),$$

**FIGURE 2:**
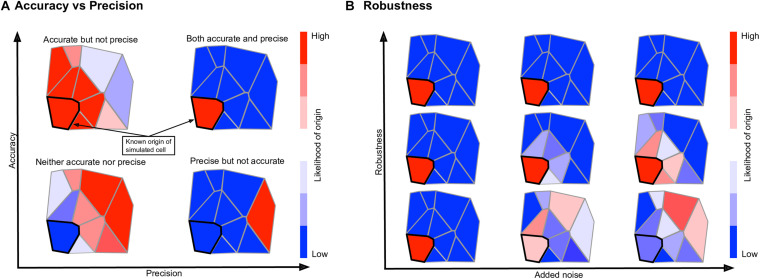
Explanation of the terms constituting the performance score. In each hypothetical mapping heatmap, the known location of the input cell is highlighted in black. **(A)** The accuracy score measures whether or not the known location receives a high likelihood; the precision score measures whether or not other locations receive low likelihoods. **(B)** The robustness score measures how much the accuracy and precision scores change if random noise is added to the input cell. A mapping method which is accurate, precise, and robust is given a high performance score while a mapping method that lacks in any or all of the three areas is given a lower performance score.

*S*_*i*,*j*_ = *S*(*c*_*i*_,*p*_*j*_) is the correspondence score of cell *c*_*i*_ to position *p*_*j*_, and *E*_*i*_ is interpreted as the error in the mapping of cell *c*_*i*_. The quantity σ^∗^ in the robustness term is calculated by determining the accuracy and precision penalty terms with no Gaussian noise added to the input data, then calculating the same two penalties with various levels of Gaussian noise with standard deviation σ ∈ [0,1]. The quantity σ^∗^ is defined to be the level of Gaussian noise required to raise the mean of the accuracy and precision penalties by 0.1 from their values with no added noise, or σ^∗^ = 1, whichever is smallest. The exponent of four in the robustness term was chosen empirically such that the robustness term does not dominate the performance score, keeping in mind that expression levels are normalized to [0,1] before calculating the correspondence scores, so e.g., σ^∗^ = 0.5 means a method requires noise on the order of half of the expression levels to raise the precision and accuracy penalties by 0.1. The performance score has a range of [0,1], where a performance score of *E=1* represents an ideal mapping that maps a cell to its known location with high confidence, to all other locations with low confidence, and does so in a manner robust to noise. An illustration of each term in the performance score is shown in Figure 2.

This performance score is limited by the fact that it relies on ground truth knowledge of the spatial origin of a single cell/spot to determine the performance of a given mapping method. However, this ground truth knowledge is not available for actual scRNA-seq data. To more directly quantify the mapping performance on actual scRNA-seq datasets, we use a measure of predictive reproducibility, obtained from a *k*-fold cross validation scheme, in which we randomly split the common genes in the reference atlas and scRNA-seq data into *k* folds and calculate the correspondence score for each method using all but one fold. The correspondence scores are then used as coefficients in a weighted sum to predict the value of the dropped-out genes in each fold for each cell (scRNA-seq predictive reproducibility) or each spatial position (atlas predictive reproducibility) and determine the error in the predicted expression level. The predicted expression of gene *k* in cell *c*_*i*_ is computed as ${\hat{c}}_{i}^{\left(k\right)}=\sum_{j=1}^{P}S_{i,j}^{\left(k\right)}\,p_{j}^{\left(k\right)}/\sum_{j=1}^{P}S_{i,j}^{\left(k\right)}$ and the predicted expression of gene *k* in position *p*_*j*_ is computed as ${\hat{p}}_{j}^{\left(k\right)}=\sum_{i=1}^{C}S_{i,j}^{\left(k\right)}\,c_{i}^{\left(k\right)}/\sum_{i=1}^{C}S_{i,j}^{\left(k\right)}$ where $S_{i,j}^{\left(k\right)}$ is the correspondence score between cell *c*_*i*_ and position *p*_*j*_ with genes in folds not containing gene *k* and $c_{i}^{\left(k\right)}$ and $p_{j}^{\left(k\right)}$ are the known expression values of gene *k* from the scRNA-seq and the spatial atlas data, respectively. To accommodate the sparsity of data, we compute the predictive reproducibility scores separately for cells or positions with zero expression values and with positive expression values. For example, we measure the predictive reproducibility for the task of reproducing gene *k* in scRNA-seq data on cells with zero expression using $R_{s\,c\,\_\,z\,e\,r\,o}^{\left(k\right)}=1-\sum_{i\in I_{s\,c\,\_\,z\,e\,r\,o}^{\left(k\right)}}\left|{\hat{c}}_{i}^{\left(k\right)}-c_{i}^{\left(k\right)}\right|/\left|I_{s\,c\,\_\,z\,e\,r\,o}^{\left(k\right)}\right|$ where $I_{s\,c\,\_\,z\,e\,r\,o}^{\left(k\right)}=\left\{i:c_{i}^{\left(k\right)}=0\right\}$. Taking the average over all common genes results in a single score *R*_*sc_ zero*_, and in the same manner, we define *R*_*sc_ nonzero*_, *R*_*atlas_ zero*_, and *R*_*atlas_ nonzero*_. When producing predictive reproducibility scores, we use the same k-fold split across all methods to ensure a fair comparison.

### Comparisons of Multiple Methods Using Simulated scRNA-seq Data

**FIGURE 3:**
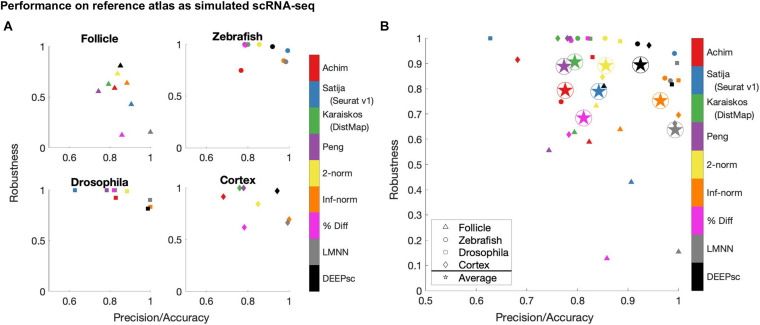
Summary of the robustness, precision, and accuracy scores of the implemented methods on four different biological systems **(A)**, as well as the simple average across all four **(B)**. These scores are each defined to be one minus the corresponding penalty term in the performance score, so that a higher score is better. Since most methods have near perfect accuracy scores, the x-axis shows a mean of the precision and accuracy scores. The y-axis shows the robustness scores for each method. Due to memory constraints, we were unable to run Seurat v1 on the cortex dataset.

**FIGURE 4:**
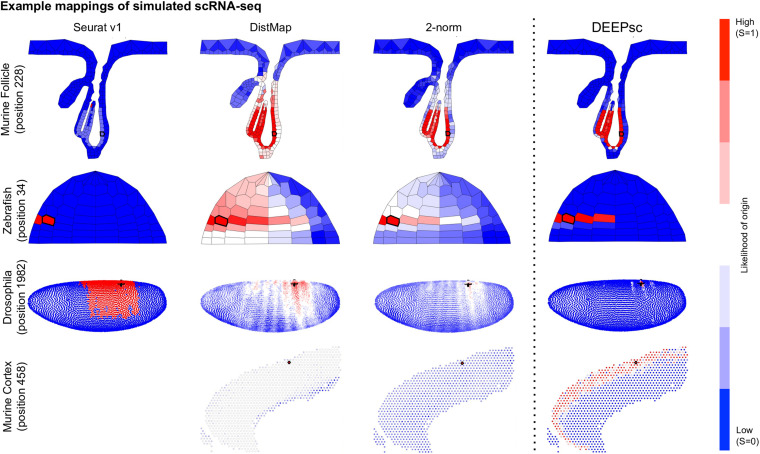
Example mappings of simulated single cells produced by various existing methods on four different biological systems, with DEEPsc mappings for comparison. The simulated input cell for the murine follicle system corresponds to position 228. For the Zebrafish system (for which Seurat was introduced), the simulated input cell corresponds to position 34. For Drosophila (for which DistMap was introduced), the simulated input cell corresponds to position 1982. For the murine frontal cortex, the simulated input cell corresponds to position 458. Each known position is highlighted in black in each of the heatmaps.

**FIGURE 5:**
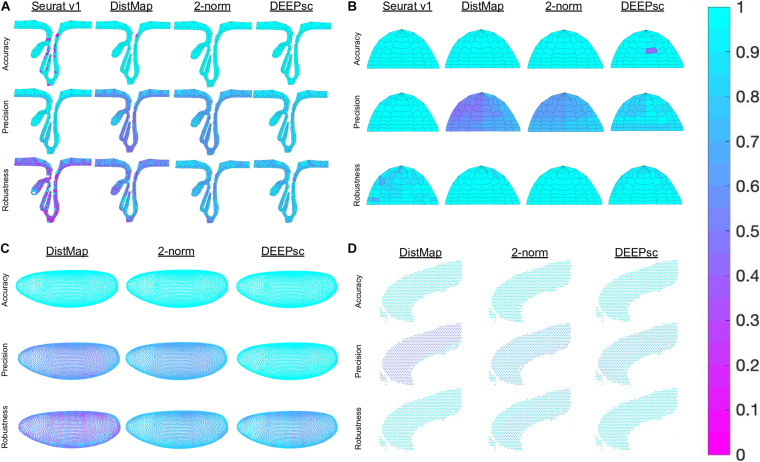
Heatmap representation of the various components of the performance score on a per position basis in **(A)** the follicle system, **(B)** the Zebrafish, **(C)** the Drosophila embryo, and **(D)** the murine frontal cortex. We were unable to run Seurat v1 on the Drosophila embryo and cortex data due to memory constraint. The penalty terms for each simulated cell, including robustness, were computed individually and plotted as a heatmap.

Using the performance score, we benchmarked the methods developed by Achim et al. (2015) and Satija et al. (2015) (Seurat v1), Karaiskos et al. (2017) (DistMap), and Peng et al. (2016) together with our DEEPsc method and applied them to four different biological systems: the zebrafish embryo (Satija et al., 2015), the *Drosophila* embryo (Karaiskos et al., 2017), the murine hair follicle (Joost et al., 2016), and the murine frontal cortex, downloaded from the 10x Genomics Spatial Gene Expression Datasets. The reference atlas for the zebrafish embryo contains the binarized expression of 47 genes on 64 spatial bins that assemble half of the hemisphere of the 6hpf embryo (Satija et al., 2015). The *Drosophila* embryo reference atlas contains 84 genes on 3,039 spatial positions (Karaiskos et al., 2017). The spatial reference atlas generated with the Visium technology (Ståhl et al., 2016) for the murine frontal cortex contains 32,285 genes on 961 spatial positions (a subset presenting the frontal cortex from the original data), from which we kept 2755 genes from the 3,000 most variable genes in spatial data that are also present in scRNA-seq data. Segmenting a standard diagram of the follicle into 233 spatial positions and using FISH imaging of eight genes identified as spatially localized (Joost et al., 2016), we manually defined a continuous reference atlas for the follicle (section “Materials and Methods”). For mapping methods requiring a binary reference atlas, we defined a cutoff expression of 0.2 to be considered on in this follicle reference atlas of follicle. We further implemented several baseline methods for benchmark comparisons, including several methods using predefined metrics where the correspondence score *S* is defined to be the 2-norm, infinity norm, or mean percent difference in the space of common genes between the input cell and spatial position. We also implemented a large margin nearest neighbor (LMNN) method that learns a linear metric (section “Materials and Methods”). Figure 3 shows a scatter plot of the penalty terms constituting the performance score of each implemented method on each of the four biological systems, as well as the average for each method across all four systems. Table 1 includes the numerical values for each penalty term, as well as the calculated performance score for each method. Figure 4 includes example heatmaps of simulated cells for each of the biological systems. The penalty terms for the individual locations are shown in Figure 5.

**TABLE 1:** Numerical values of each of the three constituent terms of the performance score, as determined from simulated scRNA-seq data for each biological system, as well as the average across all systems.

Method	Accuracy (Author)	Precision Term	Robustness Term	Performance Score
**Follicle**				
(Achim)	0.0043	0.3484	0.4116	0.7452
Seurat v1 (Satija)	0.0795	0.1076	0.5704	0.7475
DistMap (Karaiskos)	0.0043	0.4076	0.3723	0.7386
(Peng)	**0.0000**	0.5118	0.4439	0.6814
2-norm (baseline)	**0.0000**	0.3255	0.2686	0.8020
Inf-norm (baseline)	0.0005	0.2299	0.3613	0.8028
% difference (baseline)	**0.0000**	0.2829	0.8722	0.6150
LMNN (baseline)	**0.0000**	**0.0002**	0.8455	0.7181
DEEPsc (ours)	0.0272	0.2684	**0.1904**	**0.8380**
**Zebrafish**				
(Achim)	**0.0000**	0.4645	0.2516	0.7613
Seurat v1 (Satija)	**0.0000**	**0.0156**	0.0604	**0.9747**
DistMap (Karaiskos)	**0.0000**	0.3989	**0.0000**	0.8670
(Peng)	**0.0000**	0.4296	**0.0000**	0.8568
2-norm (baseline)	**0.0000**	0.2902	0.0003	0.9302
Inf-norm (baseline)	**0.0000**	0.0536	0.1588	0.9292
% difference (baseline)	**0.0000**	0.4249	0.0095	0.8552
LMNN (baseline)	**0.0000**	0.0315	0.1689	0.9332
DEEPsc (ours)	0.0339	0.1281	0.0230	0.9383
**Drosophila**				
(Achim)	**0.0000**	0.3407	0.0759	0.8611
Seurat v1 (Satija)	0.6605	0.0848	**0.0000**	0.7516
DistMap (Karaiskos)	**0.0000**	0.3496	0.0024	0.8827
(Peng)	**0.0000**	0.4313	**0.0000**	0.8562
2-norm (baseline)	**0.0000**	0.2310	0.0130	0.9186
Inf-norm (baseline)	**0.0000**	**0.0006**	0.1671	0.9441
% difference (baseline)	**0.0000**	0.3597	0.0013	0.8797
LMNN (baseline)	**0.0000**	0.0052	0.0987	**0.9653**
DEEPsc (ours)	0.0087	0.0179	0.1827	0.9303
**Cortex**				
(Achim)	**0.0000**	0.6357	0.0859	0.7594
Seurat v1 (Satija)	–	–	–	–
DistMap (Karaiskos)	**0.0000**	0.4778	**0.0000**	0.8407
(Peng)	**0.0000**	0.4400	**0.0000**	0.8533
2-norm (baseline)	**0.0000**	0.3008	0.1546	0.8482
Inf-norm (baseline)	**0.0000**	**0.0006**	0.3042	0.8984
% difference (baseline)	**0.0000**	0.4332	0.3817	0.7284
LMNN (baseline)	**0.0000**	0.0143	0.3376	0.8827
DEEPsc (ours)	**0.0000**	0.1167	0.0289	**0.9515**
**Average**				
(Achim)	0.0011	0.4473	0.2063	0.7818
Seurat v1 (Satija)	0.1850	0.0693	0.2103	0.8246
DistMap (Karaiskos)	0.0011	0.4085	**0.0937**	0.8323
(Peng)	**0.0000**	0.4532	0.1110	0.8119
2-norm (baseline)	**0.0000**	0.2869	0.1091	0.8748
Inf-norm (baseline)	0.0001	0.0712	0.2479	0.8936
% difference (baseline)	**0.0000**	0.3752	0.3162	0.7696
LMNN (baseline)	**0.0000**	**0.0128**	0.3627	0.8748
DEEPsc (ours)	0.0175	0.1328	0.1063	**0.9145**

*For each term, a value closer to zero signifies lower error. For the performance score, a value closer to one indicates a better performing method. The best method for each term is bolded for each system.*

The majority of methods were able to project the simulated scRNA-seq cells to their known spatial origins with high accuracy. Specifically, Seurat v1 and DistMap achieve high performance scores in the zebrafish embryo and Drosophila embryo datasets that they were originally applied to, respectively. Designed to be a system-adaptive method, DEEPsc has the best average performance score across the four datasets (Table 1). Moreover, while some methods are stronger in terms robustness or precision, DEEPsc attains a balance between robustness and precision (Figure 3). This balance is especially important when investigating the impact of cellular spatial neighborhood on cell fate acquisition. It is desired to narrow down the inferred spatial neighborhood (precision) and at the same time reduce the sensitivity to noise (robustness). The high precision and robustness of DEEPsc is consistently observed across all locations in the dataset (Figure 5). Finally, it is worth mentioning that DEEPsc has a significantly higher robustness in the follicle dataset which has the smallest number of genes and is the noisiest among the four datasets.

### Applications to Real scRNA-seq Datasets

**FIGURE 6:**
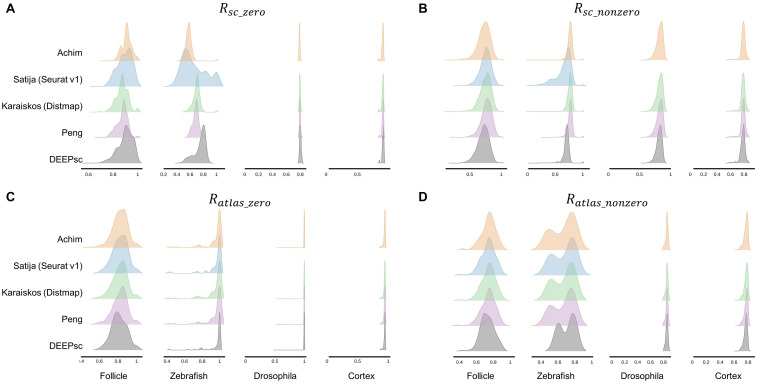
Ridgeline plots of the zero **(A)** and nonzero **(B)** scRNA-seq predictive reproducibility of individual cells in the scRNA-seq datasets and zero **(C)** and nonzero **(D)** atlas predictive reproducibility of individual positions in the spatial atlas for the four studied systems. We were unable to run Seurat v1 on the Drosophila embryo and cortex data due to memory constraints.

We now map actual scRNA-seq data for each system and calculate the predictive reproducibility for each method (Table 2 and Figure 6). For the follicle, the scRNA-seq data contains 1,422 cells with 26,024 genes measured containing the eight genes in the spatial atlas (Joost et al., 2016). For the Drosophila embryo, we used the scRNA-seq dataset with 1,297 cells and 8,924 genes among which all the 84 spatial genes are present (Karaiskos et al., 2017). For the Zebrafish embryo, there are 1,152 cells and 11,978 genes in the scRNA-seq dataset with all the 47 spatial genes included (Satija et al., 2015). For the murine frontal cortex, we used the scRNA-seq dataset provided by the Allen Institute (Tasic et al., 2016), generated with SMART-Seq2, which contains 14,249 cells and 34,617 genes, from which a set of 2,755 genes were found to be present in the top 3,000 highly variable genes in spatial atlas. These four datasets cover different situations. The follicle data contains a moderate number of locations, and the cells form well-defined layered structures such that there could be long and thin spatial regions that contain the same cells. The zebrafish embryo spatial data has a suboptimal resolution such that each spatial location consists of multiple cells. This data helps to evaluate the methods in treating coarse spatial atlases. The Drosophila embryo data contains rich spatial characteristics. There is a well-defined global ventral-dorsal/anterior-posterior coordinate system. Locally, there is also a striped pattern in the lateral side of the embryo. The frontal cortex data examines spatial gene expression at the transcriptomics level, and functions as a demonstration that DEEPsc is able to maintain a high performance on high-dimensional datasets.

**TABLE 2:** Predictive reproducibility of each method for real scRNA-seq data.

Method (Author)	Follicle	Zebrafish	Drosophila	Cortex	Average
**R_sc_zero_**					
(Achim)	**0.8772**	0.5537	0.7798	0.8019	0.7531
Seurat v1 (Satija)	0.8335	0.6842	–	–	0.7589
DistMap (Karaiskos)	0.8404	0.6641	0.7850	0.8055	0.7738
(Peng)	0.8219	0.6375	0.7859	0.8092	0.7636
Two-norm (baseline)	0.8017	0.6973	0.7874	0.8114	0.7745
Inf-norm (baseline)	0.8641	0.6180	0.7807	0.8141	0.7692
% difference (baseline)	0.8357	0.5657	0.7790	0.8079	0.7471
LMNN (baseline)	0.8254	0.6795	0.7917	0.8120	0.7772
DEEPsc (ours)	0.8344	**0.7335**	**0.7961**	**0.8165**	**0.7951**
**R_sc_nonzero_**					
(Achim)	0.7495	0.7698	0.8126	0.6693	0.7503
Seurat v1 (Satija)	0.7640	0.6975	–	–	0.7308
DistMap (Karaiskos)	0.7705	0.7619	0.8103	0.6685	0.7528
(Peng)	0.7801	0.7663	0.8114	0.6680	0.7565
Two-norm (baseline)	**0.7891**	0.7386	0.8083	0.6667	0.7507
Inf-norm (baseline)	0.7496	0.7636	**0.8128**	**0.6695**	0.7489
% difference (baseline)	0.7740	**0.7721**	0.8115	0.6690	**0.7567**
LMNN (baseline)	0.7730	0.7477	0.8117	0.6643	0.7492
DEEPsc (ours)	0.7352	0.7026	0.8080	0.6691	0.7287
**R_atlas_zero_**					
(Achim)	0.7680	0.9042	0.9264	0.8360	0.8587
Seurat v1 (Satija)	0.7681	0.9088	–	–	0.8385
DistMap (Karaiskos)	0.7674	0.9005	0.9259	0.8374	0.8578
(Peng)	0.7707	0.9006	0.9267	0.8406	0.8597
Two-norm (baseline)	0.7681	0.9003	0.9278	0.8411	0.8593
Inf-norm (baseline)	0.7623	0.9050	0.9259	0.8343	0.8569
% difference (baseline)	0.7714	0.9035	0.9261	**0.8438**	0.8612
LMNN (baseline)	0.7677	0.8937	**0.9289**	0.8359	0.8566
DEEPsc (ours)	**0.7881**	**0.9148**	0.9257	0.8415	**0.8675**
**R_atlas_nonzero_**					
(Achim)	0.7598	0.6658	0.8523	0.5124	0.6976
Seurat v1 (Satija)	0.7570	0.6776	–	–	**0.7173**
DistMap (Karaiskos)	0.7584	0.6709	0.8527	0.5127	0.6987
(Peng)	0.7570	0.6682	0.8530	**0.5135**	0.6979
Two-norm (baseline)	0.7582	0.6755	0.8530	0.5135	0.7001
Inf-norm (baseline)	0.7583	0.6745	0.8534	0.5130	0.6998
% difference (baseline)	0.7573	0.6669	0.8524	0.5134	0.6975
LMNN (baseline)	0.7573	0.6764	**0.8564**	0.5129	0.7008
DEEPsc (ours)	**0.7724**	**0.7079**	0.8527	0.5125	0.7114

*A value closer to one signifies higher predictive reproducibility. A missing entry signifies that we were not able to run the relevant method on the given dataset. The best method for each term is bolded for each system.*

For the baseline models, we linearly normalized each gene in the log-normalized scRNA-seq dataset onto the interval [0,1]. Continuous spatial atlases with expression values in the [0,1] range were used for the follicle, Drosophila embryo, and murine frontal cortex systems, the latter two having been linearly normalized to [0,1] in the same fashion as the scRNA-seq data. Since a continuous spatial atlas for Zebrafish embryo is lacking, we applied a spatial convolution to the binary atlas and added a small amount of Gaussian noise to simulate a continuous atlas. The 2-norm, Inf-norm, percent difference, and LMNN baseline models are then applied to the vectors of the commonly expressed genes in the spatial atlas and scRNA-seq data. For DEEPsc, we first applied a PCA reduction to the spatial atlas, and then applied the same linear transformation to the normalized expression values of the common genes in the scRNA-seq data. The feature vectors for the locations in the spatial atlas and the cells in the scRNA-seq data in the PCA space were then fed to the neural network. For the four existing methods, we followed the procedure as described in the associated original publications, scaling the resulting correspondence scores to [0,1] for direct comparison with baseline methods. For all the methods, we compute the predictive reproducibility by iterating over all common genes, attempting to reconstruct the expression of one gene using the k-fold cross validation scheme described in the previous section. We used *k=4* for the follicle and Drosophila embryo dataset, and *k=5* for the zebrafish embryo and cortex dataset.

**FIGURE 7:**
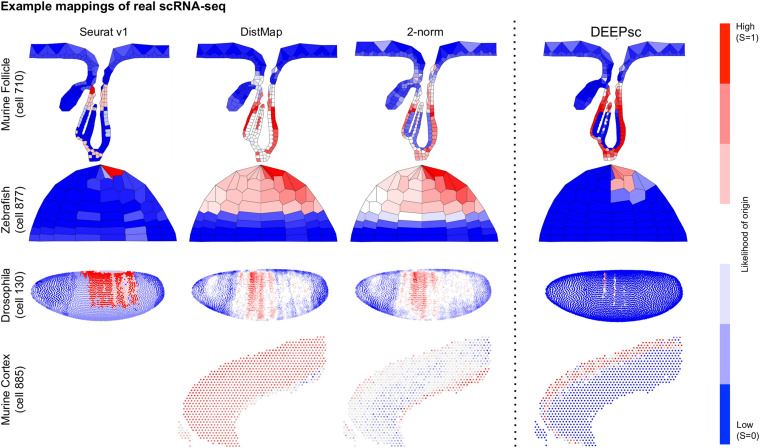
Example mappings of real single cells produced by various existing methods on four different biological systems, with DEEPsc mappings for comparison. The input cell for the murine follicle system is cell 710 from the Joost dataset. For the Zebrafish system (for which Seurat v1 was introduced), the input cell is cell 877 from the scRNA-seq dataset (Satija et al., 2015). For Drosophila (for which DistMap was introduced), the input cell is cell 130 from the scRNA-seq dataset (Karaiskos et al., 2017). For the murine frontal cortex, the input cell is cell 885 from the Allen reference dataset (Tasic et al., 2016).

DEEPsc has a comparable accuracy compared to other methods, and it also has a consistent performance across different systems (Table 2 and Figure 6). This consistent performance further demonstrates the system-adaptive advantage of DEEPsc and the benefit of using adaptive metrics over predefined ones. We also notice that similar to the simulated case, DEEPsc also achieves a balance between precision and robustness in the case of real scRNA-seq data. For example, while it exhibits high precision by mapping the example cell to a specific local spot in the Zebrafish embryo or a local strip in Drosophila embryo, it also robustly maps a cell to the entire outer bulge of the follicle instead of only part of it (Figure 7). The high precision ensures that we can resolve the heterogeneity in the spatial environment and further relate them to the heterogeneity in cell fates. The high robustness prevents the identification of false correlations. Overall, DEEPsc achieves a high predictive reproducibility across all cells in the scRNA-seq dataset.

### Comparison of Dimensionality Reduction Methods

**FIGURE 8:**
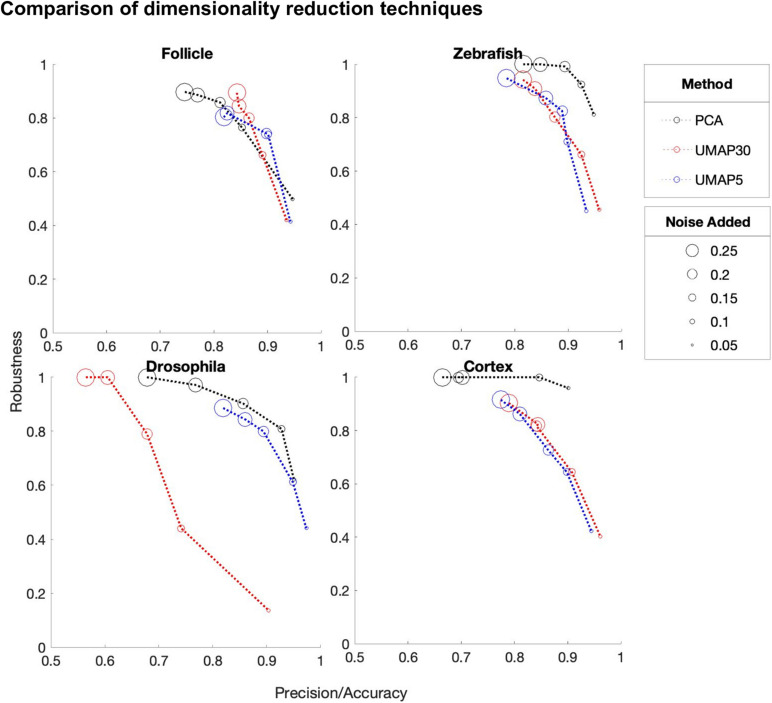
A comparison of the performance of DEEPsc networks using different dimensionality reduction methods on each of the biological systems for various levels of added noise during training. We compare principal component analysis (PCA) to Uniform Manifold Approximation and Projection (UMAP) with n_neighbors = 30 (UMAP30) and n_neighbors = 5 (UMAP5). Each of these methods reduce the dimensionality of the initial dataset to n_dimensions = 8. These scores are each defined to be one minus the corresponding penalty term in the performance score, so that a higher score is better. Since most methods have near perfect accuracy scores, the x-axis shows a mean of the precision and accuracy scores. The y-axis shows the robustness scores for each method.

Dimension reduction is a crucial initial step of DEEPsc. A dimension reduction method that can be trained on one dataset and deterministically applied to another is needed due to the separated training and predicting steps. Here, we explore two different representative dimension reduction methods in the linear and nonlinear categories, PCA and Uniform Manifold Approximation and Projection (UMAP; McInnes et al., 2018). To compare these two methods, we trained several networks with varying amounts of added noise on the reference atlases of the four studied biological systems (Figure 8). We compared PCA (8 principal components), UMAP30 (n_components = 8, n_neighbors = 30), and UMAP5 (n_components = 8, n_neighbors = 5). While on the follicle system all three reduction methods performed virtually identically, on all three other systems PCA outperformed the other reduction methods by achieving a higher robustness score while maintaining similar accuracy.

## Discussion

We have developed the DEEPsc framework, which trains a deep neural network using the known expression levels of a small subset of genes in a spatial context, then imputes that spatial information onto a non-spatial scRNA-seq dataset. Instead of using a predefined metric, DEEPsc finds a metric adaptive to data. This framework is system-adaptive and designed to be robust to noise. DEEPsc consistently performs at or above the level of several existing methods across all four biological systems studied herein, including systems for which existing methods were originally developed (Figure 3 and Tables 1, 2), based on our comprehensive performance measure. While DEEPsc achieves comparable accuracy and precision to other methods, it is significantly more robust to noise.

The source of DEEPsc’s ability to perform well across multiple biological systems is likely the generality of its neural network architecture and the multiple checks for robustness employed during training on the reference atlas. The various parameters for training a DEEPsc network, though chosen empirically, appear to translate to multiple systems effectively, so we expect DEEPsc to continue to perform well across more biological systems in future study.

One notable weakness of DEEPsc is the significant amount of training time required to produce a final mapping. While most existing reference atlas methods simply involve a deterministic calculation to produce a mapping, DEEPsc requires an initial training, and the training time depends on the number of locations in the spatial atlas. The training process of DEEPsc can be effectively accelerated by iterating over a subset of possible location pairs. Due to the dimension reduction step, DEEPsc can still be trained efficiently on datasets with large amount of genes, for example, the spatial transcriptomics data on the murine frontal cortex. Though the predefined metrics including the 2-norm and inf-norm perform well in terms of accuracy and precision, they are less robust to noise. This is further the case for LMNN as it tries to amplify any small variations. This drawback in robustness is mitigated by DEEPsc by controlling the balance between precision and robustness.

Learning a metric from high-dimensional datasets can be generally useful for analysis and integration of omics datasets. A future research interest is to decrease training time in such framework by developing a better method for reducing the size of the training set to a small, targeted fraction of relevant examples, particularly for very large atlases such as those derived from spatial transcriptomics assays. Since the size of the training set can increase quadratically with the number of positions in the atlas, it is beneficial to develop a more efficient training pipeline. We have developed a method of sparsifying the training set (section “Materials and Methods”), so that its size only increases linearly with the number of positions in the atlas, though further improvement may be warranted. The largest atlas studied here was that of Drosophila (*P=3039*), the training of which took several hours even with the sparsified training set. Typical numbers of distinct spatial locations in a spatial transcriptomics dataset can be orders of magnitude larger.

DEEPsc aside, the performance score we have created can serve as a comprehensive measure of mapping performance for future work. The performance score is able to be calculated for any mapping method that assigns a likelihood of origin from each spatial location, particularly within the reference atlas framework. It is not dependent on any specific system or mapping method, and the individual terms which constitute it allow for a detailed analysis and comparison of various methods. Potential improvements might include incorporating some amount of spatial awareness into the calculation. Currently each spatial position is treated as completely independent from every other spatial position, so the precision term, for example, can yield unintuitive results if a method maps a cell, for example, with high probability to two positions on opposite sides of a system and low probability everywhere else, compared to a different method mapping the same cell with high probability to five positions in a tightly clustered, spatially compact region of the system. If, for example, the various correspondence scores for each position with high probability were weighted by their physical distance from other cells with high probability, this term might more accurately reflect the intuitive idea of precision. Other improvements might include simplifying the calculation of the robustness term to require fewer intensive calculations.

## Conclusion

DEEPsc achieves an accuracy comparable to several existing methods while attaining improved precision and robustness. It also has a more consistent performance across the four different biological systems tested thanks to the system-adaptive design. As spatially resolved gene expression data becomes more readily available, our method will serve as a useful tool to infer spatial origins from non-spatial scRNA-seq data.

Additionally, our comprehensive performance score and the collection of reproductions of previously developed methods in a single software framework will serve as useful tools for future comparisons of spatial mapping methods. This systematic approach to imputing spatial information to scRNA-seq data is crucial to studying the spatial impact on cell fate dynamics.

## Materials and Methods

### Data Preparation for DEEPsc

Given a matrix of scRNA-seq read counts where each row is a different gene and each column is a different cell, and a matrix representing a spatial reference atlas where each row is a different gene and each column is a different spatial position, we first select common genes by eliminating rows in each corresponding to genes not in the other matrix. Once we have eliminated genes not in common, we are left with a number of cells (*C*) × number of genes (*G*) matrix for the scRNA-seq data and a number of positions (*P*) × number of genes (*G*) matrix for the spatial reference atlas.

We then apply dimensionality reduction to the atlas in the form of a PCA projection, selecting a user-configurable number of principal components to serve as feature vectors. We find in our analysis that keeping the top eight principal components yields satisfactory results. The same PCA coefficients are used to project the scRNA-seq matrix into these principal components. After projection, both matrices are normalized by dividing by the largest element in each, so that the elements are all in [0,1].

For the comparisons in section “Comparison of Dimensionality Reduction Methods,” we use the UMAP implementation by Meehan et al. (2021), found on the MATLAB Central File Exchange at https://www.mathworks.com/matlabcentral/fileexchange/71902. Specifically, we ran the run_umap() function on the spatial reference atlas with n_dimensions = 8 and n_neighbors = 30 or n_neighbors = 5 for UMAP30 and UMAP5, respectively.

### Training a DEEPsc Network

To train the DEEPsc network, we use the spatial position feature vectors themselves as simulated scRNA-seq data. The training data is a set of P^2^ vectors of length *2N*, where *N* is the reduced dimensionality of the reference atlas. The first *N* components correspond to a feature vector of one position in the reference atlas (functioning as a simulated cell) and the last *N* components correspond to some other position in the reference atlas. Each simulated cell is compared pairwise with every position in the spatial reference atlas; if the simulated cell is an exact match to a given position, the target output is chosen to be 1 (a high likelihood of origin), and if the simulated cell and chosen position are not an exact match, the target output is chosen to be 0 (a low likelihood of origin).

The DEEPsc architecture is an artificial neural network with *2N* inputs, two fully connected hidden layers with N nodes each and a single node in the output layer. Sigmoid activation functions are attached to each node, including the output node, so that the resulting output is in [0,1] and can be interpreted as a likelihood that the input cell originated from the input spatial position. To preserve robustness and avoid overfitting the training data, a layer of Gaussian noise is added to the simulated cells so that the network is pushed to learn complex nonlinear relationships among the spatial positions in the reference atlas rather than simply activate when an exact match is encountered. This Gaussian noise layer allows the user to configure the standard deviation of the added noise, as well as to configure the probability that any noise will be added in a given training epoch. We find empirically that a noise level of about 0.10 and a probability of 0.5 yield reasonable robustness to noise, though this may vary from system to system.

Since the training data will naturally consist of many more non-matches than matches, and thus the target data will contain many more zeros than ones, we use a novel custom objective function,

$$L\,\left(Y,T\right)=\sum_{i=1}^{P}{\left(y_{i}-t_{i}\right)}^{2}\,\frac{1}{1.001-t_{i}}$$

where *y*_*i*_ is the network’s predicted output and *t*_*i*_ is the target output (*t*_*i*_ = 1 if exact match and *t*_*i*_ = 0 if not), to more heavily penalize the network when it gives a false negative (low likelihood when it should be high) than when it gives a false positive (high likelihood when it should be low). This acts to counteract the tendency of the network to “learn” to simply return 0 for every single input and “ignore” any comparably rare training data with *t*_*i*_ = 1.

To further account for the sparsity of exact matches in the training set, we split it into a test and validation set, the former consisting of a configurable fraction of the inputs corresponding to exact matches as well as a configurable multiple of the inputs corresponding to non-matches. If *trainFrac = 0.9* and *trainingMultiple = 99*, for example, 90% of the exact matches will be added to the training set and 99x more non-matches will be added, so that the exact matches make up 1% of the training set. The rest of the inputs are reserved for the (generally much larger) validation set. This is beneficial in reducing training time because it allows us to train with a much smaller fraction of the *P*^2^ input vectors, giving preference to the exact matches. Indeed, this reduces the size of the actual training set to scale linearly with the size of the atlas rather than quadratically.

Training is performed in MATLAB using the *trainNetwork()* function in the Deep Learning Toolbox (The Mathworks, Inc, 2019a), for which we implemented the above-described custom network layers. Since the input data is already normalized in preprocessing, we disable the default normalization of *trainNetwork().* We use the default Glorot (Xavier and Yoshua, 2010) initialization of weights and biases in the fully connected layers. We then train each network for a maximum of 50,000 epochs of standard gradient descent with a learning rate of η=0.01, shuffle the order of the data each epoch, and use the ADAM optimization method (Kingma and Ba, 2014) with the default parameters β_1_ = 0.9, β_2_ = 0.999, and *ε* = 10^−8^. In addition to the custom objective function layer we describe above, *trainNetwork()* by default adds an *L*^2^-regularization term to the loss with a regularization factor of λ=0.0001. We monitor the RMSE of the validation set throughout training and manually stop training if it is no longer improving before the maximum number of epochs has been reached. The *trainNetwork()* function also allows for parallel computation via the Parallel Computing Toolbox (The Mathworks, Inc, 2019b), which is highly recommended but not strictly required for training.

### Creating a Reference Atlas for the Murine Follicle

To create a spatial reference atlas for the murine follicle system, we patterned the spatial coordinates of each position in the atlas off of a standard diagram of a mouse follicle found in Figure 1 of Joost et al. (2016). We constructed a Voronoi diagram around each of the cell centers and made manual adjustments to the vertices as we saw fit aesthetically. We then selected the eight genes in the atlas from the systematic staining catalog made available by Joost. We chose the genes based on a combination of high image quality and spatial diversity. Gene expression levels in [0,1] were chosen manually to best represent the images, though to eliminate any implicit bias we also added a small level of Gaussian noise to the atlas. For all methods requiring a binary atlas, we chose a cutoff of 0.2 to represent “on” expression in this atlas.

### Large Margin Nearest Neighbor Baseline

To implement a LMNN baseline for benchmarking comparison, we used code from the MATLAB Toolbox for Dimensionality Reduction found at https://lvdmaaten.github.io/drtoolbox/ and modified it for our uses. Specifically, we used the *lmnn()* function in the “techniques” subfolder, and modified the code to set *mu = 1*, i.e., to remove the “pull” term, as well as setting the number of targets to 1 (the point itself) and treating all other points as imposters. Further, we modified the slack variables to enforce a minimum separation of $\sqrt{D}$, where *D* is the dimensionality of the space (*D=G* for our applications). For the numerical experiments of the LMNN method with the cortex dataset, a PCA dimension reduction (50 PCs) was performed before applying LMNN to accommodate the large number of genes.

## Data Availability Statement

The original data used in this paper can be accessed through the following links: (1) zebrafish embryo spatial data: downloaded from (https://www.dropbox.com/s/ev78jelev0jgu5s/seurat_files_zfin.zip?dl=1) (Satija et al., 2015); (2) zebrafish embryo scRNA-seq data: GEO accession codes: GSE66688 (Satija et al., 2015); (3) Drosophila embryo spatial and scRNA-seq data: accessible at the Dream Single cell Transcriptomics Challenge through Synapse ID (syn15665609) (Karaiskos et al., 2017); (4) mouse frontal cortex spatial data: downloaded from 10x Genomics Spatial Gene Expression Datasets (https://support.10xgenomics.com/spatial-gene-expression/datasets/1.1.0/V1_Mouse_Brain_Sagittal_Anterior); (5) mouse frontal cortex scRNA-seq data: downloaded from (https://www.dropbox.com/s/cuowvm4vrf65pvq/allen_cortex.rds?dl=1) (Tasic et al., 2016); (6) follicle scRNA-seq data and spatial imaging data from which the reference atlas was derived: downloaded from the supplementary of the associated publication (Joost et al., 2016). All software developed for the purposes of this comparison are made freely available at: https://github.com/fmaseda/DEEPsc.

## Author Contributions

FM carried out computer implementation and data analysis. ZC and QN supervised the project. FM and ZC wrote the original manuscript. All authors conceived and designed the work, interpreted the simulation results, and contributed to the writing of the final manuscript.

## Conflict of Interest

The authors declare that the research was conducted in the absence of any commercial or financial relationships that could be construed as a potential conflict of interest.
